# Fingolimod-induced decrease in heart rate may predict subsequent decreasing degree of lymphocytes

**DOI:** 10.1038/s41598-018-34797-7

**Published:** 2018-11-06

**Authors:** Tokunori Ikeda, Tatsuyuki Kakuma, Mari Watari, Yukio Ando

**Affiliations:** 1 Department of Neurology, Graduate School of Medical Sciences, Kumamoto University, Kumamoto, Japan; 2 Department of Clinical Investigation, Kumamoto University Hospital, Kumamoto, Japan; 3 Department of Biostatistics Center, Kurume University, Kurume, Japan

**Keywords:** Neuroimmunology, Multiple sclerosis

## Abstract

Here, we determined whether degree of decreased heart rate due to fingolimod treatment correlates with decreasing degree of lymphocytes in relapse-remitting multiple sclerosis (RRMS). In total, 30 patients with RRMS were treated with 0.5 mg fingolimod and their heart rate recorded every 30 minutes for 24 hours. Time trends of heart rate were characterised as three individual amplitudes and phase angles from three cosine curves using a mixed-effect model. Spearman’s correlation coefficient and regression analysis were used to determine the effect of heart rate information on change in lymphocyte count pre- and post-fingolimod treatment. Moreover, the degree of decreased lymphocytes induced by fingolimod treatment on heart rate was compared between low and high influence groups. Positive correlation between amplitude from the second curve and difference in lymphocyte number (*p* = 0.006) was observed. Regression analysis was also significant (*p* = 0.002). Moreover, the second curve derived from the high amplitude group exhibited a greater decrease in lymphocyte number after fingolimod treatment than the low amplitude group (*p* < 0.001). We suggest that the degree of decreased lymphocytes after fingolimod treatment (main effect) may be predicted by estimating the influence of degree in heart rate (side effect).

## Introduction

Fingolimod is an effective oral medicine for relapse-remitting multiple sclerosis (RRMS)^1,2^. Fingolimod is a structural analog of sphingosine and mainly acts as a functional antagonist at sphingosine-1-phosphate 1 (S1P1) receptors^3,4^. Fingolimod is phosphorylated *in vivo* and binds to S1P1 receptors on leukocytes, especially lymphocytes, which retains the lymphocytes in secondary lymphoid tissue^5^. Consequently, lymphopenia in peripheral blood is induced and maintained in a steady state 1 month later^6–8^. Moreover, fingolimod has other side effects, e.g., decreased heart rate, which can present because of S1P1 receptor activation on cardiomyocytes^9^. Therefore, with initial administration of fingolimod, the appearance of excessive decreases in heart rate or abnormal arrhythmia are monitored in patients at hospital admission. Thus, it is possible to obtain heart rate information within hours of fingolimod treatment.

In the present study, we assumed a similar mechanism for decreased heart rate and leukopenia, and determined whether the degree of decrease in heart rate correlates with decreasing number of leukocytes, with a specific focus on lymphocytes.

## Results

### Statistical model of heart rate

**Figure 1:**
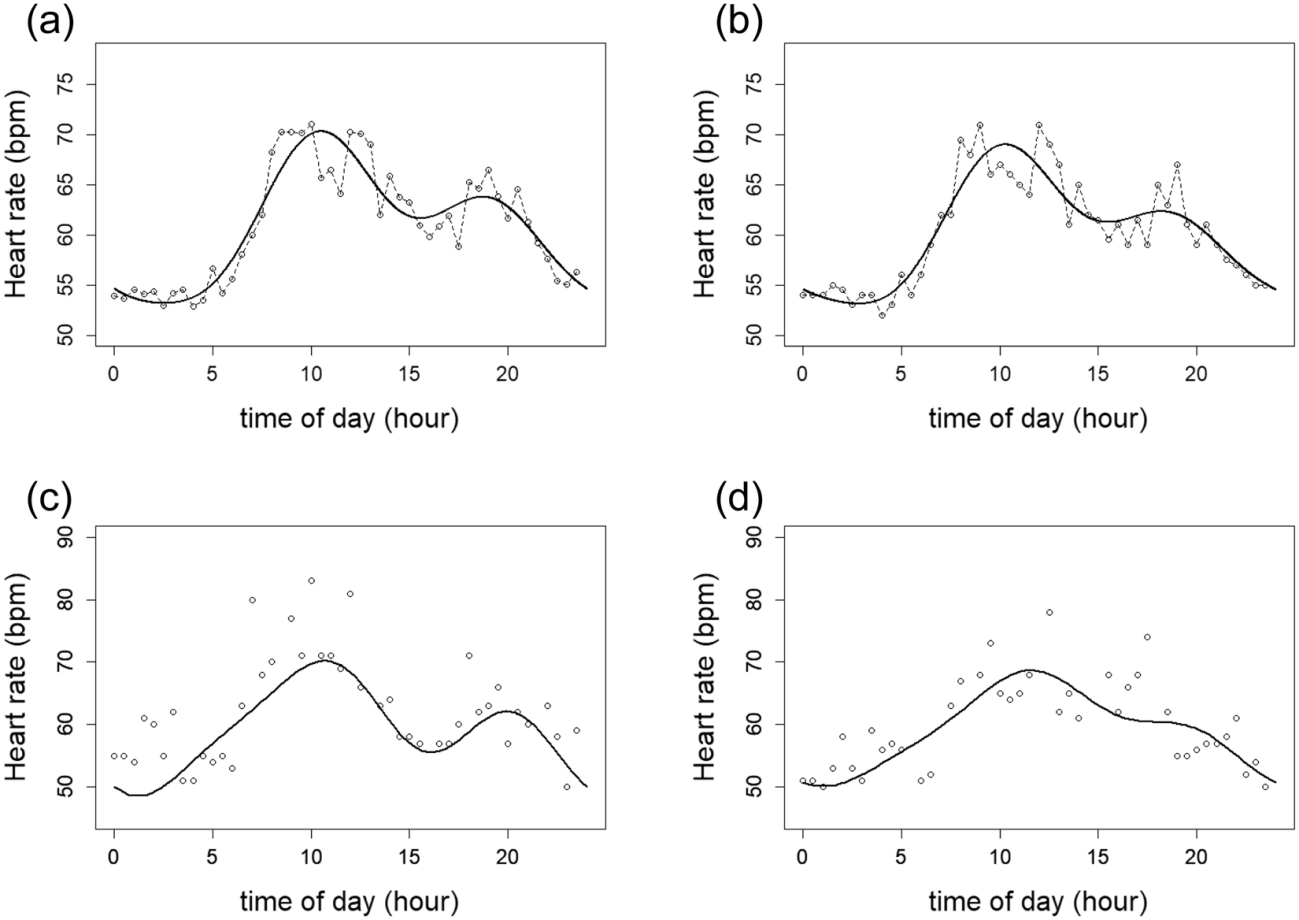
Model of circadian heart rate rhythm using three cosine curves. Mean values (**a**) and median values (**b**) of heart rate were expressed using a combination of three cosine curves, as described in the Methods. Heart rate curves for each patient were estimated using a mixed-effect model adjusted for age and sex. Representative patients shown include those with a visible (**c**) and not visible (**d**) second wave. Each dot, dotted line, and solid line represents heart rate at a specific time point, with a connected line from actual heart rate and estimated line from three cosine curves model.

To represent 24 hours of continuous heart rate after fingolimod treatment, we used a combination of three cosine curves. Our results showed correct expression of the statistical heart rate model (Fig. 1a,b). Next, heart rate curves for each patient were estimated using a mixed-effect model with three cosine curves and adjustments for age and sex. Accordingly, the shape of individual curves can be roughly classified into two groups based on whether the second wave is clearly visible or not (Fig. 1c,d).

### Correlation between heart rate and lymphocytes

**Figure 2:**
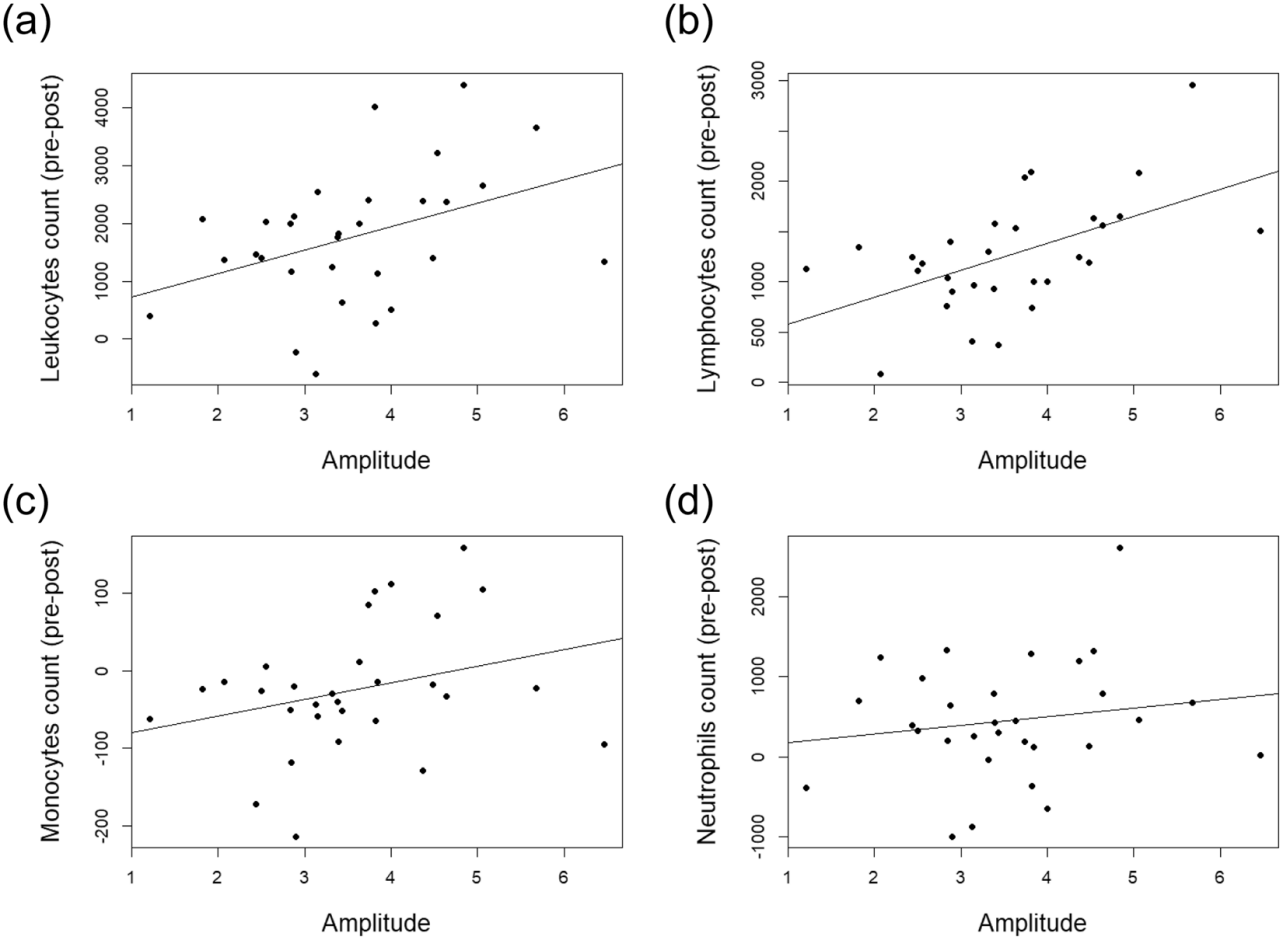
Linear regression plot. A linear regression plot showing the relationship between difference in number of pre-post leukocytes (**a**), lymphocytes (**b**), monocytes (**c**), and neutrophils (**d**) and amplitude from the second cosine curve.

For each individual, we obtained information in the form of three amplitudes and phase angles for individual predicted curves. We then examined Spearman’s correlation coefficients between amplitude or phase angle and difference in leukocyte or subset (lymphocyte, monocyte, and neutrophil) count pre- and post-fingolimod treatment (Table 1). Strong correlation was detected between differences in leukocyte and lymphocyte (0.74, *p* < 0.001) or neutrophil (0.77, *p* < 0.001) count. In addition, moderate correlation was detected between difference in leukocyte and monocyte number (0.47, *p* = 0.009). Positive correlation was confirmed between amplitude from the second cosine curve and difference in lymphocyte number (r = 0.49, *p* = 0.006). While there was weak correlation with leukocytes (r = 0.36, *p* = 0.05), monocytes (r = 0.31, *p* = 0.09), but not neutrophils (r = 0.08, *p* = 0.69). In contrast, there was virtually no correlation between other heart rate information and difference in leukocyte or subset counts, except for correlation between phase angle from the first cosine curve and difference in monocyte number (−0.61, *p* < 0.001). Next, we used regression analysis (Fig. 2 and Table 2) to examine differences in values for leukocytes (*p* = 0.028, 95% confidence interval [95%CI]: 62.24–754.93) and lymphocytes (*p* = 0.002, 95%CI: 112.45–426.72) in relation to amplitude from the second curve. To confirm this regression model, we used bootstrap testing (*n* = 50,000), and corroboratively identified the same tendency (leukocytes, *p* < 0.001, 95%CI: 332.46–1043.49; lymphocytes, *p* = 0.004, 95%CI: 78.41–347.30). There was a weak relationship between difference in value for monocytes and amplitude from the second cosine curve (*p* = 0.12, 95%CI: −4.65–47.25). In this regard, bootstrap testing confirmed a positive relationship between difference in monocytes and amplitude from the second curve (*p* = 0.045, 95%CI: 1.80–54.20). A relationship with neutrophils was not confirmed by either test (regression, *p* = 0.39, 95%CI: −131.47–345.99; bootstrap, *p* = 0.38, 95%CI: −137.16–364.32).

**Table 1:** Spearman’s correlation coefficients.

	Leukocytes	Lymphocytes	Monocytes	Neutrophils	Amplitude *A*	Amplitude *B*	Amplitude *C*	Phase angle *ψ*_1_	Phase angle *ψ*_2_	Phase angle *ψ*_3_
Leukocytes	1.00	0.74	0.47	0.77	0.15	0.36	0.04	−0.33	0.07	0.00
Lymphocyte	0.74	1.00	0.41	0.36	0.11	0.49	0.09	−0.19	0.14	0.19
Monocytes	0.47	0.41	1.00	0.35	−0.20	0.31	−0.1	−0.61	0.05	0.15
Neutrophils	0.77	0.36	0.35	1.00	0.07	0.08	0.06	−0.13	0.12	−0.03
Amplitude *A*	0.15	0.11	−0.20	0.07	1.00	0.00	0.07	−0.05	−0.04	−0.26
Amplitude *B*	0.36	0.49	0.31	0.08	0.00	1.00	0.03	−0.17	0.18	0.28
Amplitude *C*	0.04	0.09	−0.1	0.06	0.07	0.03	1.00	0.20	0.59	0.12
Phase angle *ψ*_1_	−0.33	−0.19	−0.61	−0.13	−0.05	−0.17	0.20	1.00	0.04	0.07
Phage angle *ψ*_2_	0.07	0.14	0.05	0.12	−0.04	0.18	0.59	0.04	1.00	0.38
Phage angle *ψ*_3_	0.00	0.19	0.15	−0.03	−0.26	0.28	0.12	0.07	0.38	1.00

Leukocytes, lymphocytes, monocytes, and neutrophils represent the difference in number pre- and post-fingolimod treatment. Amplitude (*A*, *B*, and *C*) and phase angle (*ψ*_1_, *ψ*_2_, and *ψ*_3_) represent amplitude and phase angle from the first, second, and third cosine curves, respectively.

**Table 2:** Results of multivariate regression analysis.

Endogenous variable	Exogenous variable	Estimate	SE	*p*-Value	95% CI
Leukocytes	Intercept	312.00	659.00	0.64	(−979.70, 1603.62)
	Amplitude *B*	408.60	176.70	0.028	(62.24, 754.93)
Lymphocytes	Intercept	307.85	299.00	0.31	(−278.18, 893.87)
	Amplitude *B*	269.59	80.17	0.002	(112.45, 426.72)
Monocytes	Intercept	−100.59	49.38	0.051	(−197.37, −3.81)
	Amplitude *B*	21.30	13.24	0.12	(−4.65, 47.25)
Neutrophils	Intercept	71.32	454.25	0.88	(−818.99, 961.62)
	Amplitude *B*	107.26	120.80	0.39	(−131.47, 345.99)

Abbreviations: SE = standard error; CI = confidence interval. Leukocytes, lymphocytes, monocytes and neutrophils represent the difference in number pre- and post-fingolimod treatment. Amplitude *B* represents the amplitude from the second cosine curve.

### Comparison of difference in lymphocyte number in low and high amplitude groups

**Figure 3:**
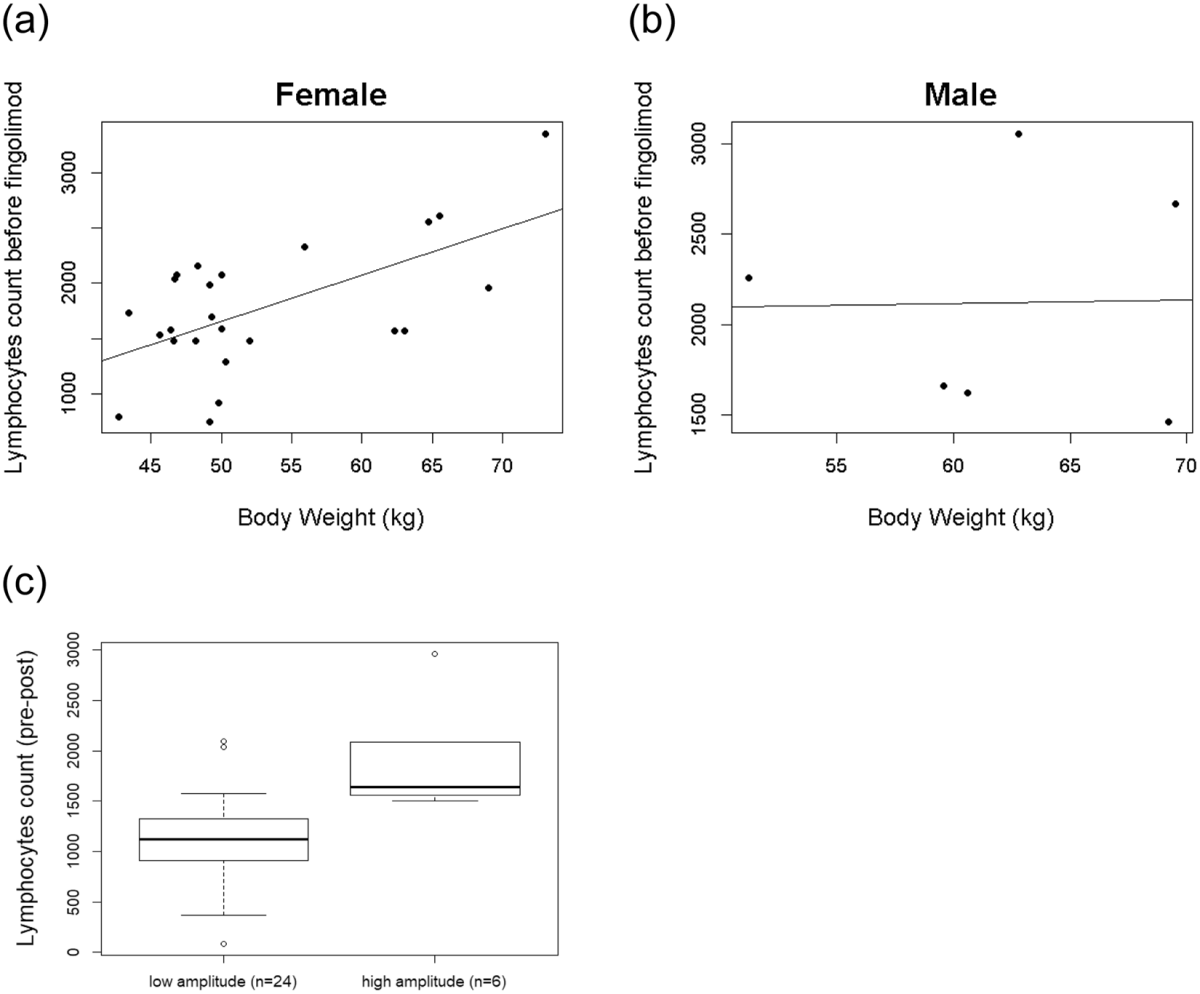
Linear regression plot showing the relationship between lymphocyte count and body weight (kg) in females (**a**) and males (**b**). Comparison in decreased lymphocyte number by classification of low and high amplitude groups. To distinguish patients with low or high sensitivity for fingolimod, a cut-off value was determined by decision tree analysis using the amplitude value from the second curve. Amplitude <4.53 was designated as the low amplitude group and ≥4.53 was the high amplitude group. Boxplot comparing the difference in lymphocyte number between both groups. Wilcox rank sum test was performed (**c**).

Underweight women are reported to be at risk for low lymphocytes^10^. Accordingly, we examined correlation and regression between body weight and lymphocyte number before fingolimod treatment. We found weak correlation (r = 0.39, *p* = 0.06) and a strong linear relationship (*p* = 0.002, 95%CI: 19.04–64.88) between these two variables in females (Fig. 3a) but not males (correlation, r = 0.09, *p* = 0.92; linear relationship, *p* = 0.97, 95%CI: −90.55–94.45) (Fig. 3b). Thus, our findings support the assumption that underweight women are in general at latent risk for low lymphocyte number. Finally, to discriminate between difference in lymphocyte number in individuals showing higher and lower effectiveness for fingolimod, we performed decision tree analysis using amplitude from the second cosine curve and a decided cut-off value. As shown in Fig. 3c, the high amplitude group (cut-off value ≥ 4.53) showed a greater decrease in lymphocyte number than the low amplitude group (*p* < 0.001, 95%CI: −1238.00–−317.58). We also examined body weight in the high amplitude group (*n* = 6) and found that all the patients were female but not necessarily the lightest in weight.

Because baseline lymphocyte count before fingolimod treatment was different in each individual, we next performed analysis of covariance (ANCOVA) and adjusted lymphocyte count number before fingolimod treatment between both groups. Accordingly, lymphocyte count number before fingolimod treatment was related to decreased lymphocyte number, as previously reported^10^ (Table 3). Moreover, our analysis showed decreased lymphocyte number in the high amplitude group compared with the low amplitude group (*p* = 0.015, 95%CI: 70.27–506.88) (Table 3).

**Table 3:** Results of analysis of covariance.

Endogenous variable	Exogenous variable	Estimate	SE	*p*-Value	95% CI
Lymphocytes	Intercept	−216.44	135.74	0.12	(−482.48, 49.60)
	Pre-lymphocytes	0.77	0.07	<0.001	(0.63, 0.92)
	Low amplitude group (ref)				
	High amplitude group	288.57	111.38	0.015	(70.27, 506.88)

Abbreviations: SE = standard error; CI = confidence interval; ref = reference. Lymphocytes represent the difference in number pre- and post-fingolimod treatment. Pre-lymphocytes represent lymphocyte count number before fingolimod treatment. Low or high amplitude groups were determined based on amplitude from the second cosine curve.

## Discussion

Lymphopenia after fingolimod treatment is the desired outcome for therapy of RRMS, yet it is possible that an excessive decrease of lymphocytes increases the risk of developing infection. Therefore, a marker is needed that can predict an excessive decrease of lymphocytes after fingolimod treatment. Previously, patients with a low lymphocyte number before fingolimod treatment and underweight women were prone to lymphopenia after fingolimod treatment^11^. In addition, underweight women are at risk for low lymphocytes^10^, yet the incidence rate of females with MS is higher than males^12^. Therefore, the risk of excessive lymphopenia must be considered when administering fingolimod to underweight women with RRMS.

Additionally, in this study, we focused on the functional mechanism of fingolimod. Fingolimod is a functional antagonist for four of five S1P receptors (namely S1P1, S1P3, S1P4, and S1P5)^13–15^. S1P1 and S1P3 receptors are expressed on cardiac myocytes, on which fingolimod promotes activation of G-protein-coupled inwardly rectifying potassium (GIRK) channels^16,17^. Although S1P3 receptors are involved in the fingolimod-induced decrease in heart rate, a novel S1P receptor modulator, siponimod (BAF312) (which influences S1P1 and S1P5 but not S1P3) was previously shown to induce a decrease in heart rate. Subsequently, considerable involvement of S1P1 receptors are recognised in this decreased heart rate^18,19^. In fact, S1P1 receptor expression levels are higher in cardiac myocytes than S1P3 receptors^17^. Further, half maximal effective concentration (EC50) in binding of S1P1 receptors and phosphorylated fingolimod is low compared with S1P3 receptors^20^. In contrast, no clinical adverse effects on cardiac rhythm have been reported with a selective S1P1 receptor modulator, amiselimod (MT-1303)^21,22^. Although the reason for the discrepancy in these results is not fully understood, amiselimod shows low activation of GIRK channels on cardiac myocytes compared with fingolimod^22^. Altogether, it is more likely that S1P1 receptors on cardiac myocytes are a key player in the decrease of heart rate, at least regarding fingolimod. Leukocytes are mainly composed of neutrophils, monocytes, and lymphocytes, and these cell types also express S1P receptor subtypes, including S1P1 receptors^5,23–29^. Decreased circulating lymphocytes and monocytes due to fingolimod or siponimod treatment involves S1P1 receptors^23,25^. However, the influence of fingolimod on circulating neutrophils is weak, and S1P4 and S1P1 receptors are the main S1P receptor subtypes in neutrophils^27–29^. Thus, S1P1 receptors are also involved in development of leukopenia via fingolimod.

Fingolimod binds S1P1 receptors, which are internalised through repetitive stimulation by fingolimod^30^. As a result, cardiac myocytes escape the influence of fingolimod to signal downstream, with subsequent early recovery of heart rate rhythm despite the presence of fingolimod. Without fingolimod treatment, S1P1 receptors on lymphocytes are naturally internalised by high concentration of physiological ligand (i.e., S1P) in blood and efferent lymph^30^. However, because internalisation of S1P1 receptors is reversible, expression of S1P1 receptors is restored in secondary lymphoid tissue (e.g., lymph nodes), in which S1P concentration is at a low density^30,31^. When lymphocytes migrate from secondary lymphoid tissue to efferent lymph, they utilise the density gradient of S1P between secondary lymphoid tissue (low concentration) and efferent lymph (high concentration)^30^. In contrast, with fingolimod treatment, fingolimod concentration is high in secondary lymphoid tissue, which induces S1P1 receptor internalisation on lymphocytes and interferes with lymphocyte migration into efferent lymph. Therefore, fingolimod retains lymphocytes in secondary lymphoid tissue and a decreased lymphocyte count persists in blood, unlike in cardiac myocytes^32,33^.

Nonetheless, there is a time lag between decreased heart rate and lymphocyte count, regardless of the mechanism induced by fingolimod via S1P1 receptors^7^. Our hypothesis for this is based on the finding that a decreasing heart rate by fingolimod is reportedly caused by sinus node cells in cardiac myocytes^34^. While total cardiac myocyte number is estimated at approximately 2–3 × 10^9^ cells^35^, sinus node cell number is not known. However, the sinus node area is approximately 13.5 ± 2.5 mm in length, 1.2 ± 0.3 mm in height, and 5.6 ± 1.4 mm in width, with a low occupancy in heart tissue^36^. Therefore, it is highly likely that the number of sinus node cells is small. Furthermore, total cell number of lymphocytes is approximately 2 × 10^12^ cells in adults^26^. In addition, there are 400–600 lymph nodes (secondary lymphoid tissue) distributed throughout the body, and lymphocytes circulate within secondary lymphoid tissue, blood and efferent lymph, unlike sinus node cells which are fixed^37^. Because the body contains many secondary lymphoid tissues, the time for fingolimod distribution may be different in each secondary lymphoid tissue. In addition, the usual state of circulating lymphocytes and presence of larger number of lymphocytes may affect the probability that fingolimod encounters S1P1 receptors on lymphocytes. Therefore, although the decrease in peripheral lymphocyte count starts at an early stage, similar to the influence for sinus node cells (which are smaller in number and exist as a solid organ), it may take time to reach the steady state decrease in lymphocyte count induced by fingolimod treatment. Actually, lymphocyte count in blood gradually decreases in a day-dependent manner to reach a steady state^38^. In addition, decreased lymphocyte count in blood begins at 4–5 hours after fingolimod administration in mice^4^. Moreover, deceasing degree of pulse rate in a seated position and lymphocyte count after fingolimod administration shows dose-dependency^7,8^.

Finally, the mechanisms underlying a decrease in heart rate via fingolimod are assumed to be similar to leukopenia. Therefore, we determined whether the degree of decreased heart rate by fingolimod correlates with later decreasing degree of leukocytes or its subsets (including lymphocytes, monocytes, and neutrophils).

To validate this, we accurately aggregated continuous heart rate information among individuals, and then isolated shapes from certain variables of this data. In this respect, although the spline model can easily collate continuous heart rate information, it cannot appropriately isolate heart rate information as variables. Hence, the circadian rhythm of heart rate after fingolimod treatment was expressed by cosine curves using a mixed-effect model, with amplitude and phase angle for each of the three cosine curves obtained^39,40^. By this means, continuous heart rate information was summarised as minimal variables formed by three amplitudes and phase angles. In addition, we used a mixed-effect model to account for differences in medication time of fingolimod in each individual (which are evident as differences in behaviour or fluctuation for each heart rate). Usually a mixed-effect model analyses repeated measurement data (such as heart rate in our data), and contains random effects to consider variations in each subject^41^.

Hence, we confirmed whether any variables are linked to differences in number of leukocytes or subsets pre- and post-fingolimod treatment. Accordingly, we identified a positive relationship between amplitude from the second cosine curve and difference in lymphocyte number. A similar tendency was observed for leukocytes and monocytes but not neutrophils. Each of these relationships corresponded to the degree of S1P1 receptor expression in each subset, as described above. Altogether, these results suggest that amplitude from the second cosine curve is most important. Because the heart rate wave naturally presents as a monomodal wave, we assumed that the influence of fingolimod treatment could be extracted from the second wave in a fingolimod-influenced bimodal wave. Therefore, each amplitude and phase angle from the first and second cosine curves were mainly represented as first and second waves of the bimodal wave, respectively. Moreover, amplitude and phase angle from the third cosine curve acted as modulators of the bimodal wave. Actually, although we employed three cosine curve models, by reference to Akaike’s information criterion and expectation, as modulators of the third cosine curve for the bimodal wave, amplitude from the second cosine curve in the two cosine curve model also manifested a similar outcome as the three cosine curve model. Hence, we interpreted the importance of amplitude from the second cosine curve based on the following rationale. When fingolimod strongly affects cardiac myocytes (sinus node cells), the fingolimod-related decrease in heart rate reaches its lowest point within 6 hours^7^, which is represented by a deep trough between the first and second waves of the bimodal wave. After that, because the body attempts to restore heart rate to its natural state, we inferred that amplitude from the second cosine curve increases depending on the trough depth, with a high amplitude reflecting the strength of fingolimod’s effect. Certainly, in our data, the high amplitude group had a value of ≥4.53 in the second cosine curve, which was exhibited by a decreased difference in lymphocyte number pre- and post-fingolimod treatment. Moreover, we used 24, 12, and 8 hours in our model as each respective cycle of the three cosine curves. Accordingly, the second cosine curve has a cycle of 12 hours and half time of 6 hours, which is the lowest point of this cycle. Therefore, our statistical model suggests a decreasing heart rate peak within 6 hours of fingolimod treatment.

Otherwise, we also expected that fingolimod wholly impacted on the circadian rhythm of heart rate, and that the power of this influence is apparent from the first wave of the bimodal wave, namely, lower amplitude from the first cosine curve. However, this was not observed. In this regard, there was a negative relationship between phase angle from the first cosine curve and difference in monocyte number. Although the reason for this is not obvious, earlier appearance of the second wave in the bimodal wave (which indicates a strong effect of fingolimod) affected duration of the first wave. Consequently, a shorter phase angle might occur, and monocytes may be sensitive to this phenomenon.

In our analysis, six patients were treated with interferon (IFN)-β at induction of fingolimod treatment. Because IFN-β decreases lymphocyte number at considerable frequency^42^, lymphocyte count before fingolimod treatment may affect IFN-β in these patients. However, this situation is a usual occurrence in daily medical practice, and analyses must be performed by applying real-world situations. In addition, because we used ANCOVA as the statistical model for adjusting baseline lymphocyte count, the clinical state before fingolimod treatment was considered.

Above all, although our study has limitations regarding the number of RRMS patients, it is possible that degree of decreased lymphocytes after fingolimod treatment (main effect) may be predicted by estimating the influence of degree in amplitude from the second cosine curve (side effect). If so, by monitoring heart rate for 24 hours after fingolimod treatment, neurologists may be able to use amplitude information to detect an excessive influence of fingolimod on the main effect. Consequently, an earlier return visit may be considered for fingolimod-treated RRMS patients to check the degree of decreased lymphocytes.

S1P modulator drugs for MS that do not influence heart rate will be available in the future. However, this will take time; therefore MS patients should be treated as occasionally having an advantageous side effect without simply considering an adverse event.

## Methods

### Patients

In total, 30 patients with RRMS were evaluated. They were administered 0.5 mg fingolimod once daily at Kumamoto University Hospital (Kumamoto, Japan) between 2012 and 2016 (Table 4). Of these patients, fingolimod was taken around 9 am or 1 pm in 24 and 6 patients, respectively. Six patients switched from IFN-β therapy to fingolimod, while the other patients were not administered any disease-modifying drugs (including IFN-β) at induction of fingolimod. During initial treatment of fingolimod, all patients underwent 24 hours of continuous electrocardiogram (ECG) monitoring in hospital, with heart rate recorded every 30 minutes in the resting state. The number of leukocytes and its subsets (i.e., lymphocytes, monocytes, and neutrophils) were measured at a central clinical laboratory of Kumamoto University Hospital. This study was approved by the Institutional Review Board of Kumamoto University (Permit Number: 1279). Informed consent was obtained from all patients for participation in this study, and all experiments were performed in accordance with the Declaration of Helsinki.

**Table 4:** Characteristics in MS patients.

	MS patients
	n = 30
Age, median (IQR)	36.00 (30.25, 44.00)
Male, *n* (%)	6 (20.00)
Leukocytes before fingolimod, median (IQR)	5425.00 (4840.00, 6375.00)
Leukocyte after fingolimod, median (IQR)	3617 (3205.00, 5010.00)
Lymphocytes before fingolimod, median (IQR)	1677.00 (1496.00, 2138.00)
Lymphocytes after fingolimod, median (IQR)	523.90 (402.60, 656.20)
Monocytes before fingolimod, median (IQR)	330.20 (253.10, 4020.30)
Monocytes after fingolimod, median (IQR)	333.10 (284.70, 382.80)
Neutrophils before fingolimod, median (IQR)	3157.00 (2663.00, 3850.00)
Neutrophils after fingolimod, median (IQR)	2566.00 (2192.00, 3730.00)
Amplitude *A*	7.14 (5.43, 8.15)
Amplitude *B*	3.42 (2.85, 4.28)
Amplitude *C*	2.79 (1.99, 4.55)
Phase angle *ψ*_1_	1.40 (−0.25, 2.05)
Phage angle *ψ*_2_	−2.06 (−2.61, 2.62)
Phage angle *ψ*_3_	0.91 (0.25, 1.69)

Abbreviations: MS = multiple sclerosis; HD = healthy donor; IQR = interquartile range. Leukocytes, lymphocytes, monocytes and neutrophils represent the difference in number pre- and post-fingolimod treatment. Amplitude (*A*, *B*, and *C*) and phase angle (*ψ*_1_, *ψ*_2_, and *ψ*_3_) represent amplitude and phase angle from the first, second, and third cosine curves, respectively.

### Statistical analysis

A retrospective longitudinal observational study was performed at Kumamoto University Hospital. Sample size was determined with consideration of the number of inpatients to Kumamoto University Hospital during the survey period. Shapiro–Wilk’s test was performed to identify normal distribution of variables. Heart rate manifested as circadian rhythms, as described previously^39^. To investigate the relationship between 24 hours of heart rate monitoring data and leukocyte or subset (lymphocyte, monocyte, and neutrophil) count during the treatment period, two analytical steps were performed. Repeated measures of heart rate were treated as a predicting variable, while change in leukocyte or subset count was modelled as the response variable. In the first step, time trend of heart rate data was modelled as a circadian rhythm. Because circadian rhythms statistically express cosine curves^40^, amplitude and phase angle characterised the time trend of heart rate, and were predicted for each patient based on the mixed-effect model as follows:

$$\begin{array}{rcl}{y}_{ij} & = & {\beta }_{0}+{A}_{i}\,\cos \,(\tfrac{2\pi ({t}_{ij}-{\psi }_{1i})}{P})+{B}_{i}\,\cos \,(\tfrac{4\pi ({t}_{ij}-{\psi }_{2i})}{P})+{C}_{i}\,\cos \,(\tfrac{6\pi ({t}_{ij}-{\psi }_{3i})}{P})\\  &  & +\,{\beta }_{1}{x}_{1i}+{\beta }_{2}{x}_{2i}+{\varepsilon }_{ij}\\  & = & {\beta }_{0}+{\alpha }_{i}\,\cos \,(\tfrac{2\pi {t}_{ij}}{P})+{\beta }_{i}\,\sin \,(\tfrac{2\pi {t}_{ij}}{P})+{\gamma }_{i}\,\sin \,(\tfrac{4\pi {t}_{ij}}{P})\\  &  & +\,{\delta }_{i}\,\sin \,(\tfrac{4\pi {t}_{ij}}{P})+{\epsilon }_{i}\,\sin \,(\tfrac{6\pi {t}_{ij}}{P})+{\theta }_{i}\,\sin \,(\tfrac{6\pi {t}_{ij}}{P})\\  &  & +\,{\beta }_{1}{x}_{1i}+{\beta }_{2}{x}_{2i}+{\varepsilon }_{ij}\\ {\alpha }_{i} & = & {A}_{i}\,\cos \,(\tfrac{2\pi {\psi }_{1i}}{P}),{\beta }_{i}={A}_{i}\,\sin \,(\tfrac{2\pi {\psi }_{1i}}{P}),{A}_{i}=\sqrt{{\alpha }_{i}^{2}+{\beta }_{i}^{2}},{\psi }_{1i}=\tfrac{P}{2\pi }\,\arctan \,(\tfrac{{\beta }_{i}}{{\alpha }_{i}})\\ {\gamma }_{i} & = & {B}_{i}\,\cos \,(\tfrac{4\pi {\psi }_{2i}}{P}),{\delta }_{i}={B}_{i}\,\sin \,(\tfrac{4\pi {\psi }_{2i}}{P}),{B}_{i}=\sqrt{{\gamma }_{i}^{2}+{\delta }_{i}^{2}},{\psi }_{2i}=\tfrac{P}{4\pi }\,\arctan \,(\tfrac{{\delta }_{i}}{{\gamma }_{i}})\\ {\epsilon }_{i} & = & {C}_{i}\,\cos \,(\tfrac{6\pi {\psi }_{3i}}{P}),{\theta }_{i}={C}_{i}\,\sin \,(\tfrac{6\pi {\psi }_{3i}}{P}),{C}_{i}=\sqrt{{\epsilon }_{i}^{2}+{\theta }_{i}^{2}},{\psi }_{3i}=\tfrac{P}{6\pi }\,\arctan \,(\tfrac{{\theta }_{i}}{{\epsilon }_{i}})\end{array}$$

*y*_*ij*_: Value of heart rate at a time (hour) in each individual. *β*_0_: Common intercept. *π*: Circle ratio. *P*: 24 hours. *x*_1*i*_: Age (year) in each individual. *x*_2*i*_: Sex of each individual. Male was used as the base. ${\alpha }_{i},{\beta }_{i},{\gamma }_{i},\,{\delta }_{i},\,{{\epsilon }}_{i},{\theta }_{i}$: Total estimated coefficient value from fixed and random effects in the mixed-effect model. *ψ*_1*i*_: Individual phase angle of the first cosine curve at peak time in each individual. *ψ*_2*i*_: Individual phase angle of the second cosine curve at peak time in each individual. *ψ*_3*i*_: Individual phase angle of the third cosine curve at peak time in each individual. *t*_*ij*_: Time point of day (hour) in each individual. *A*_*i*_: Individual amplitude of first cosine curve. *B*_*i*_: Individual amplitude of second cosine curve. *C*_*i*_: Individual amplitude of third cosine curve. *ε*_*ij*_: Residual error (which followed a normal distribution).

In this model, age and sex affect heart rate; therefore these two variables were used as adjusted factors^43^. In addition, heart rate >90 bpm was regarded as an outlier (from the perspective of normal heart rate i.e., resting state) and was excluded from the model. In this mixed model, three types of cosine and sine curves showed a random effect. Three amplitudes (*A*_*i*_, *B*_*i*_, and *C*_*i*_) and phase angles (*ψ*_1*i*_, *ψ*_2*i*_, and *ψ*_3*i*_) were calculated in each individual. In some evaluations, mean or median values from all MS patients were used to express the above three cosine curves. In the second step, Spearman’s correlation coefficients and a regression model were used to examine the effect of heart rate on change in leukocyte or subset counts. The components of age and sex for heart rate information were included in the mixed-effect model, and were therefore not used in the regression model. Because change in leukocytes or subsets was stable after 1 month of fingolimod treatment^7,8^, difference in leukocyte and subset count was used. This was calculated by the difference in two mean values from 1 year to the period immediately before fingolimod treatment, and from 1 month to 1 year after fingolimod treatment. In addition, decision tree analysis was used to identify the cut-off value for determining whether amplitude was related to decreased lymphocyte number. The Wilcoxon rank sum test was performed to compare the difference in lymphocyte number in low and high amplitude groups. Difference in lymphocyte number in these two groups was performed by ANCOVA based on leukocyte count before fingolimod treatment. Analyses were performed using R version 3.3.2 (The R Foundation for Statistical Computing, Vienna, Austria) and SAS Version 9.4 (SAS Institute Inc., Cary, North Carolina, USA), with the level of statistical significance set at *p* < 0.05.

## Data Availability

The datasets generated during and/or analysed during the current study are available from the corresponding author on reasonable request.
