# Author Correction: Fingolimod-induced decrease in heart rate may predict subsequent decreasing degree of lymphocytes

**DOI:** 10.1038/s41598-019-48630-2

**Published:** 2019-08-28

**Authors:** Tokunori Ikeda, Tatsuyuki Kakuma, Mari Watari, Yukio Ando

**Affiliations:** 1 Department of Neurology, Graduate School of Medical Sciences, Kumamoto University, Kumamoto, Japan; 2 Department of Clinical Investigation, Kumamoto University Hospital, Kumamoto, Japan; 3 Department of Biostatistics Center, Kurume University, Kurume, Japan

Correction to: *Scientific Reports* 10.1038/s41598-018-34797-7, published online 06 November 2018

This Article contains an error in the definition of *y*_*ij*_ in the Statistical Analysis section of the Methods.

$$\begin{array}{rcl}{y}_{ij} & = & {\beta }_{0}+{A}_{i}\,{\rm{co}}s(\frac{2\pi ({t}_{ij}-{\psi }_{1i})}{P})+{B}_{i}\,{\rm{co}}s(\frac{4\pi ({t}_{ij}-{\psi }_{2i})}{P})+{C}_{i}\,{\rm{co}}s(\frac{6\pi ({t}_{ij}-{\psi }_{3i})}{P})\\  &  & +{\beta }_{1}{x}_{1i}+{\beta }_{2}{x}_{2i}+{\varepsilon }_{ij}\\  & = & {\beta }_{0}+{\alpha }_{i}\,\cos (\frac{2\pi {t}_{ij}}{P})+{\beta }_{i}\,\sin (\frac{2\pi {t}_{ij}}{P})+{\gamma }_{i}\,\sin (\frac{4\pi {t}_{ij}}{P})+{\delta }_{i}\,\sin (\frac{4\pi {t}_{ij}}{P})\\  &  & +{{\epsilon }}_{i}\,\sin (\frac{6\pi {t}_{ij}}{P})+{\theta }_{i}\,\sin (\frac{6\pi {t}_{ij}}{P})+{\beta }_{1}{x}_{1i}+{\beta }_{2}{x}_{2i}+{\varepsilon }_{ij}\end{array}$$

should read:

$$\begin{array}{rcl}{y}_{ij} & = & {\beta }_{0}+{A}_{i}\,{\rm{co}}s(\frac{2\pi ({t}_{ij}-{\psi }_{1i})}{P})+{B}_{i}\,{\rm{co}}s(\frac{4\pi ({t}_{ij}-{\psi }_{2i})}{P})+{C}_{i}\,{\rm{co}}s(\frac{6\pi ({t}_{ij}-{\psi }_{3i})}{P})\\  &  & +{\beta }_{1}{x}_{1i}+{\beta }_{2}{x}_{2i}+{\varepsilon }_{ij}\\  & = & {\beta }_{0}+{\alpha }_{i}\,\cos (\frac{2\pi {t}_{ij}}{P})+{\beta }_{i}\,\sin (\frac{2\pi {t}_{ij}}{P})+{\gamma }_{i}\,\cos (\frac{4\pi {t}_{ij}}{P})+{\delta }_{i}\,\sin (\frac{4\pi {t}_{ij}}{P})\\  &  & +{{\epsilon }}_{i}\,\cos (\frac{6\pi {t}_{ij}}{P})+{\theta }_{i}\,\sin (\frac{6\pi {t}_{ij}}{P})+{\beta }_{1}{x}_{1i}+{\beta }_{2}{x}_{2i}+{\varepsilon }_{ij}\end{array}$$

